# Do Editorial Policies Support Ethical Research? A Thematic Text Analysis of Author Instructions in Psychiatry Journals

**DOI:** 10.1371/journal.pone.0097492

**Published:** 2014-06-05

**Authors:** Daniel Strech, Courtney Metz, Hannes Knüppel

**Affiliations:** 1 Hannover Medical School, CELLS – Centre for Ethics and Law in the Life Sciences, Institute for History, Ethics and Philosophy of Medicine, Hannover, Germany; 2 Leibniz University of Hannover, CELLS – Centre for Ethics and Law in the Life Sciences, Department of Philosophy, Hannover, Germany; University of Louisville, United States of America

## Abstract

**Introduction:**

According to the Declaration of Helsinki and other guidelines, clinical studies should be approved by a research ethics committee and seek valid informed consent from the participants. Editors of medical journals are encouraged by the ICMJE and COPE to include requirements for these principles in the journal’s instructions for authors. This study assessed the editorial policies of psychiatry journals regarding ethics review and informed consent.

**Methods and Findings:**

The information given on ethics review and informed consent and the mentioning of the ICMJE and COPE recommendations were assessed within author’s instructions and online submission procedures of all 123 eligible psychiatry journals. While 54% and 58% of editorial policies required ethics review and informed consent, only 14% and 19% demanded the reporting of these issues in the manuscript. The TOP-10 psychiatry journals (ranked by impact factor) performed similarly in this regard.

**Conclusions:**

Only every second psychiatry journal adheres to the ICMJE’s recommendation to inform authors about requirements for informed consent and ethics review. Furthermore, we argue that even the ICMJE’s recommendations in this regard are insufficient, at least for ethically challenging clinical trials. At the same time, ideal scientific design sometimes even needs to be compromised for ethical reasons. We suggest that features of clinical studies that make them morally controversial, but not necessarily unethical, are analogous to methodological limitations and should thus be reported explicitly. Editorial policies as well as reporting guidelines such as CONSORT should be extended to support a meaningful reporting of ethical research.

## Introduction

According to the Declaration of Helsinki, research studies should 1) be approved by an independent research ethics committee (REC) and 2) seek informed consent (IC) from the participants [1]. These principles have in turn been addressed by the International Committee of Medical Journal Editors (ICMJE) and the Committee on Publication Ethics (COPE). Both groups publish core requirements for editing and reporting research findings. For example the ICMJE state in their recommendations (previously known as *uniform requirements for manuscripts*) that “the requirement for informed consent should be included in the journal’s Instructions for Authors. When informed consent has been obtained, it should be indicated in the published article”. The COPE code of conduct asks editors to ensure that reports of clinical trials cite compliance with the Declaration of Helsinki (DoH), Good Clinical Practice, and other relevant guidelines on safeguarding participants. Editors are encouraged by the ICMJE and COPE to apply and distribute these guidelines [2], [3]. Consequential responsibilities of journal editors have been widely discussed [4]–[9].

However, empirical data from several studies throughout the last two decades suggest insufficient reporting of ethics review approvals and IC procedures in peer-reviewed articles and meta-analysis [10]–[18]. Weil and colleagues demonstrated that only 52% of the articles in paediatric journals reported ethical approval and one in seven studies had not undergone REC review [15].

A few studies have assessed journals’ instructions to authors on the reporting of ethical issues, but none has done so in the field of psychiatry, and no study so far has investigated both the instructions given to authors on the journals’ websites and those given during the submission process [6], [19]–[21]. Furthermore, editorial policies on more specific reporting of ethical approval or informed consent have not yet been assessed systematically. For example, more specific reporting might be expected with regard to how the capacity to give informed consent was assessed in patients with Alzheimer or schizophrenia [17], [22]. More specific reporting on ethics review might be expected with respect to the justification of studies in which patients, for example, (i) receive placebos, (ii) are withdrawn from standard medication, (iii) undergo “wash out” phases or (iv) are administered a challenge agent [22], [23].

The objective of this study was to assess the editorial policies of a representative sample of psychiatry journals on the reporting of ethics review and informed consent in original research papers. Furthermore, this study assessed whether and how psychiatry journals refer to international guidelines on publication ethics.

## Methods

Based on Journal Citation Reports [24] from 2011 we identified 130 journals indexed in the subject category “psychiatry”. We further specified a subsample of 10 psychiatry journals with the highest impact factor (“TOP-10”). We restricted our analysis to journals published in English or German. We accessed the ‘author’s instructions’ or similar texts on the journals’ websites between July and August 2012. We further accessed the instructions given during the online submission procedure in January 2013. The online submission procedures were entered by a fake submission of an ‘original paper’, or a ‘clinical research’ or ‘clinical trial’ paper. All PDFs or website texts were downloaded using WinHTTrack 3.46-1 for documentation. The membership of all journals of COPE or ICMJE was checked on the respective web pages (www.publicationethics.org and www.icmje.org) in August 2013.

We assessed if and how the DoH, ICMJE and COPE were mentioned in the author instructions or during the submission procedure. Further we assessed the information given on ethics review, and informed consent. We had three rating options: 1) “not mentioned”, 2) “information recommended” or 3) “information required”. The rating “information recommended” was applied to moderate wording in the author instructions such as “should” or “we recommend that…” The rating “information required” was applied to strong wording like “authors must…”, “we expect authors to…” or “we require authors to…” Particular specifications and requirements on ethics approval and informed consent were extracted and recorded.

Multiple designations of the responsible ethical authority was treated as referring to the same body (ethical review board, ethical review committee, research ethics board or institutional review board).

In this text we use the term “research ethics committee” (REC) consistently.

Two authors (HK, CM) independently assessed the editorial policies and then merged their findings. Inconsistent findings were discussed in consultation with a third member of the group (DS).

We calculated frequency data using standard descriptive statistics.

## Results

After excluding 7 journals which were not in English or German, or had no web page, we included 123 journals in our analysis (116 in English and 7 in German).

### Information and Requirements Regarding International Guidelines on Publication Ethics

Of the 123 psychiatry journals, 46 (37%) referred to the Declaration of Helsinki in the author instructions or during their online submission process. Sixty-eight (55%) of all journals referred to the ICMJE but of these only 11 (17%) were listed as “following URM” (now: “following ICMJE recommendations”) on *icmje.org*. Conversely, while 28 (33%) of all journals referred to COPE in the author instructions or during their online submission process, 62 (50%) were indicated as signed up to COPE on *publicationethics.org*.

From the TOP-10 psychiatry journals (ranked by impact factor) 20% referred to the Declaration of Helsinki, 90% to the ICMJE’s URMs (now: ICMJE recommendations), and 20% to COPE. Fifty percent were listed as “following URM” (now: “following ICMJE recommendations”) on the ICMJE website and 60% as signed up on the COPE website.

### Information and Requirements Regarding Ethics Review

Of the 123 psychiatry journals, 66 (54%) recommended or required ethics review explicitly in their author instructions or during their online submission process, but only 17 (14%) required that REC approval must be mentioned in the manuscript (table 1). Further specifications or additional requirements on the reporting of ethics review information were made by 20 (16%) of all 123 journals. Twelve (10%) required the reporting of the REC’s name and seven (6%) an original document for REC approval. No editorial policy asked for justifications from the principal investigator or the ethics review board with respect to particular risks and ethical concerns in the research design (e.g. the need to withdraw a patient’s standard medications or administration of a challenge agent that can provoke psychiatric symptoms). The findings for the TOP-10 journals have the same tendency (table 1).

**Table 1:** Detailed information about the statements on ethics review in the authors instructions or during the submission process.

Ethics review	Wording examples according to the ratings	All journals	Top-10 journals
		N = 123	N = 10
Recommended or Required ethics review		66 (54%)	5 (50%)
Specified their requirements concerning the statement		27 (22%)	3 (30%)
Publication of theinformation in themanuscript	“For human or animal experimental investigations, appropriate institutional review board approval is required and should be described in the Methods section of the paper.”	17 (14%)	2 (20%)
Required to name theREC	“Manuscripts that involve investigations on human participants must give the name of the ethics committee that approved the study.”	12 (10%)	2 (20%)
Required originaldocuments or evidence	“An author must make available all requisite formal and documented ethical approval from an appropriate research ethics committee using humans or human tissue.”	7 (6%)	0 (0%)
Required explanation,if there was no REC approval	“State whether institutional review board approval was obtained for the investigation; if it was not, provide an explanation.”	3 (2%)	0 (0%)
Required to reportexemption or requirements from theREC	“If a study has been granted an exemption from requiring ethics approval, this should also be detailed in the Methods section.”	2 (2%)	0 (0%)
Not Recommended or Required ethics review		57 (46%)	5 (50%)

### Information and Requirements on Informed Consent

Giving the editor details of the informed consent (IC) procedure was recommended or required by 71 (58%) of all 123 journals, but only 23 (19%) required this information in the manuscript (table 2). Further specifications or additional requirements on the reporting of informed consent were made by 18 (15%) of the journals: seven (6%) asked for information on how the decision-making capacity of participants was assessed, and 5 (4%) requested to know whether the child’s assent was obtained in addition to the informed consent of the child’s proxies. Ten journals (8%) asked for the original IC templates to be provided to the editor. The ratings for the TOP-10 journals were similar (see table 2).

**Table 2:** Detailed information about the statements on informed consent (IC) in the authors instructions or during the submission process.

Informed Consent	Wording examples according to the ratings	All Journals	Top-10
		N = 123	N = 10
Recommended or Required IC		71 (58%)	8 (80%)
Specified their requirements concerning the statement		34 (28%)	4 (40%)
Publication of theinformation in themanuscript	“Within the Methods section, authors should indicate that ‘informed consent’ has been appropriately obtained and state the name of the REC, IRB or other body that provided ethical approval.”	23 (19%)	2 (20%)
Required information onthe capacity assessment	“Authors of reports on human studies should include detailed information on the informed consent process, including the method(s) used to assess the subject’s capacity to give informed consent, and safeguards included in the study design for protection of human subjects.”	7 (6%)	2 (20%)
Required information onchild’s assent	“In the case of children, authors are asked to include information about whether the child’s assent was obtained in addition to that of the legal guardian.”	5 (4%)	1 (10%)
Required originaldocuments or evidence	“An author must make available all requisite formal and documented ethical approval from an appropriate research ethics committee using humans or human tissue, including evidence of anonymisation and informed consent from the client (s) or patient (s) studied.”	10 (8%)	1 (10%)
Not Recommended or Required IC		52 (42%)	2 (20%)

Furthermore, the 71 journals that required statements on IC differed in whether and how they demanded particular issues to be addressed in the IC forms. While 58 journals asked authors in a rather general manner to “include a statement in the manuscript that informed consent was obtained” 13 (11%) journals further specified what the IC should include. The most frequent specification was that IC procedures must include a “full explanations of the procedures” (N = 9). Others state e.g. that the study subject should be informed about “possible side effects” (n = 3), “purpose of the research” (n = 1), “the right to decline to participate and to withdraw from the research once participation has begun” (n = 1), “prospective research benefits” (n = 1), “limits of confidentiality” (n = 1), or “incentives for participation” (n = 1).

## Discussion

Several international policies and guidelines aim to improve the adherence to ethical standards and responsible conduct in clinical research and its reporting. For example, the ICMJE and COPE advise medical journal editors to require information about informed consent (IC) procedures and the approval of the local research ethics committee/institutional review board (REC/IRB). A minor but nevertheless striking finding of this study is the inconsistency between the number of journals mentioning one of these organisations (ICMJE = 55% and COPE = 33%) in their editorial polices and the number of journals officially registered with these organisations (ICMJE = 9% and COPE = 50%). This inconsistency questions the seriousness of mentioning or signing up to these organisations at least for the journals that either mention or have signed up but not both.

The ICMJE recommend that the requirement for informed consent should be included in the journal’s instructions to authors, and that the published article should indicate when informed consent has been obtained. While every second editorial policy of the 123 reviewed psychiatry journals recommended or required REC approval (54%) or IC procedure (58%) in the author instructions, only a minority of journals explicitly demanded the reporting of these issues in the manuscript (14% and 19%). The TOP-10 psychiatry journals (ranked by impact factor) performed similarly in this regard. Against this background it is unsurprising that only a tiny minority of editorial policies asked for the reporting of more detailed information of particulars in the ethics review (e.g. justification of ethically challenging study designs) or in the informed consent procedures (e.g. how informed consent was obtained in participants with impaired decision making or how decision-making capacity was assessed prior to informed consent). Stocking et al. found in a review of trials on Alzheimer disease that only 8% reported that decision-making capacity was assessed specifically for the reported study and that this assessment was completed before recruitment [17].

We justify in the following paragraphs why editorial policies of psychiatry journals (as well as other general and specialty journals) should require more transparent, more consistent, and more detailed reporting regarding ethical issues of published studies.

Insufficient reporting of ethical issues within biomedical research can negatively affect how trustworthy the public judge the biomedical research community to be [25]. Public trust in the research community requires evidence that this specific community has qualities such as competence and good will which merit that trust [26]. Insufficient reporting of ethical issues may not only give the impression to the public but also to the research community itself that the ethical quality of research is judged far less important than its scientific validity. However, designing a study demands both critical reflection on relevant methodological aspects (e.g. randomisation and blinding to minimise the influence of confounding biases) and on ethical issues (e.g. fair selection of, minimising risks for and obtaining valid informed consent from trial participants) [27]. Furthermore, ideal scientific design sometimes needs to be compromised for ethical reasons.

The better established requirement to report standard methodological aspects (e.g. eligibility criteria, blinding, randomization procedures [28]) has two main consequences: First, as a direct consequence it helps editors, reviewers and readers to assess the reliability and validity of the research. Second, as an indirect consequence it signals to future authors the importance of critical reflection on methodological quality in the design and conduct of a study. Likewise, editorial policies should require reporting of pertinent ethical considerations for the following reasons: A) to allow editors, reviewers and readers to assess the ethical quality of the research, B) to foster the design and conduct of future studies that meet appropriate standards of ethical research [29], C) to raise the visibility of ethical research and thereby maintain public trust and D) to facilitate a discussion and scientific evaluation of current standards and variations in real-life research ethics.

General statements such as “the study was approved by the local IRB” or “informed consent was obtained from all study participants” clearly do not meet the above described rationale for and aims of reporting ethical issues - at least not in research involving patients with disorders that may impair decision-making capacity, such as Alzheimer disease and schizophrenia, nor in research involving interventions that pose ethical concerns (see examples above and in [22], [23]).

Against the background of the presented empirical findings and normative analysis, and in accordance with former suggestions from Franklin G. Miller et al. [22] we suggest that features of clinical studies that make them morally controversial, but not necessarily unethical, are analogous to methodological limitations. Editorial policies should be revised to support a meaningful reporting of ethical research. To reach this aim, the current COPE and URM recommendations concerning the reporting of IC and REC approval should also be revised.

Studies that have morally controversial features, such as placebo controls, symptom provocation or deception, might be dismissed as unethical unless the rationale for including such features and details of safeguards to protect research participants from harm or exploitation are explained [29].

Following this line of argumentation and adding the premise that using results of (presumably) unethical studies is (at least) morally doubtful we also recommend in accordance with Michael A. Weingarten et al. [30] to include an ethical assessment in systematic reviews of clinical trials. This recommendation should be considered in revisions of manuals for systematic reviews (Cochrane handbook [31] as well as in revisions or extension of reporting guidelines such as CONSORT [28] or PRISMA [32].

## Conclusion

Only every second psychiatry journal adheres to the ICMJE’s recommendation to inform authors about requirements for informed consent and ethics review. Furthermore, only 14% and 19% of all psychiatry journals demanded the reporting of these issues in the manuscript. The TOP-10 psychiatry journals (ranked by impact factor) performed similarly in this regard. Editors have the opportunity, the right and the competence to support ethical research by (simply) updating their policies on how to report on ethical issues in clinical research.
